# A novel protein derived from lamprey supraneural body tissue with efficient cytocidal actions against tumor cells

**DOI:** 10.1186/s12964-017-0198-6

**Published:** 2017-10-16

**Authors:** Yue Pang, Changzhi Li, Shiyue Wang, Wei Ba, Tao Yu, Guangying Pei, Dan Bi, Hongfang Liang, Xiong Pan, Ting Zhu, Meng Gou, Yinglun Han, Qingwei Li

**Affiliations:** 1 College of Life Science, Liaoning Normal University, Dalian, 116081 China; 2 Lamprey Research Center, Liaoning Normal University, Dalian, 116081 China

**Keywords:** Lamprey, LIP, Cytotoxic activity, Inflammatory, Phosphatidylserine

## Abstract

**Background:**

In previous research, we found that cell secretion from the adult lamprey supraneural body tissues possesses cytocidal activity against tumor cells, but the protein with cytocidal activity was unidentified.

**Methods:**

A novel lamprey immune protein (LIP) as defense molecule was first purified and identified in jawless vertebrates (cyclostomes) using hydroxyapatite column and Q Sepharose Fast Flow column. After LIP stimulation, morphological changes of tumor cells were analysed and measured whether in vivo or in vitro.

**Results:**

LIP induces remarkable morphological changes in tumor cells, including cell blebbing, cytoskeletal alterations, mitochondrial fragmentation and endoplasmic reticulum vacuolation, and most of the cytoplasmic and organelle proteins are released following treatment with LIP. LIP evokes an elevation of intracellular calcium and inflammatory molecule levels. Our analysis of the cytotoxic mechanism suggests that LIP can upregulate the expression of caspase 1, RIPK1, RIP3 to trigger pyroptosis and necroptosis. To examine the effect of LIP in vivo, tumor xenograft experiments were performed, and the results indicated that LIP inhibits tumor growth without damage to mice. In addition, the cytotoxic action of LIP depended on the phosphatidylserine (PS) content of the cell membrane.

**Conclusions:**

These observations suggest that LIP plays a crucial role in tumor cell survival and growth. The findings will also help to elucidate the mechanisms of host defense in lamprey.

**Electronic supplementary material:**

The online version of this article (10.1186/s12964-017-0198-6) contains supplementary material, which is available to authorized users.

## Background

The adaptive immune system of jawed vertebrates can remove exogenous pathogens or abnormal cells, primarily by cytotoxicity against target cells. In addition to antibody-dependent cellular cytotoxicity and the antibody-mediated classical complement pathway, cytotoxic T lymphocytes (CTLs) are able to kill target cells infected by viruses [1]. The ability of CTLs to kill target cells primarily depends on the amount of cytoplasmic granules, such as perforin and granzyme. They induce programmed cell death in the target cell, thus eliminating cells that have become cancerous or are infected with viruses or bacteria [2]. In previous research, we observed that lamprey antisera displayed complement dependent cytolytic effects against bacteria and tumor cells [3]. In this study, a novel protein was discovered in the supraneural body (SB) of adult lamprey that has cytotoxic activity against human MCF-7 and K562 cells. We obtained the gene encoding this novel protein, called lamprey immune protein (LIP), and examined the cytotoxic activities of LIP against a variety of cultured human cells. LIP was found to have strong cytocidal effects against various human cells in vitro and in vivo. In addition, LIP specifically bound to the plasma membrane of tumor cells and then rapidly increased the membrane permeability and dramatically altered the organelle morphologies. Our analyses of the cytocidal effects of this protein demonstrate the following: (i) LIP increases the membrane permeability and alters the organelle morphologies; (ii) evokes an elevation of intracellular calcium, and inflammatory molecule levels; (iii) LIP preferentially kills tumor cells in vitro and in vivo without damage to mice; (iv) LIP upregulates the expression of caspase 1, RIPK1, RIP3 to trigger pyroptosis and necroptosis, and (v) LIP specifically recognizes and binds to PS on the plasma membrane of tumor cells. Thus, LIP is a cell-discriminating and membrane-targeting protein that causes irreversible intracellular decay in tumor cells. Because LIP possesses highly selective cytotoxicity toward human cells and has the potential to specifically recognize and kill some classes of tumor cells, the possibility of its application in medical and biological fields is anticipated.

## Methods

### Animals and cell culture

Adult lampreys (*Lethenteron camtschaticum)* weighing 121-152 g were obtained in December 2015 from the Tongjiang Valley of Songhua River, Heilongjiang Province, China. These lampreys were kept at 10 °C in glass tanks with recirculating fresh water at Liaoning Normal University. The animal experiments were performed in accordance with the regulations of the Animal Welfare and Research Ethics Committee of the Institute of Dalian Medical University’s Animal Care protocol (Permit Number: SCXK2008-0002).

Human cells used, breast adenocarcinoma cell MCF-7, hepatocyte cancer HepG2, chronic myeloid leukemia K562 cell, leukemia T cells Jurkat were purchased from the ATCC (Manassas, VA). Cells were cultured in DMEM, RPMI-1640 supplemented with 10% FBS and 1% penicillin/streptomycin (Life Technologies).

### Cell isolation and preparation of secretion

The lampreys were dissected and then wiped with 70% alcohol. The supraneural body tissues were stripped from lampreys, and the attached muscle was carefully removed and cut into small pieces approximately 1 × 1 mm^**2**^ in area with scissors, and transferred to 25 cm^**2**^ cell culture flasks containing 30 ml 2.5% trypsin at 4 **°**C till 12 h. The cells were decanted, centrifuged at 376×g for 5 min, and transferred to L15 Leibovitz Medium containing concentrations of antibiotics (100 U/ml of penicillin sulfate and 100 μg/ml of streptomycin) without FBS, convenient for protein purification. Then, cells and cell secretions were separated by centrifugation, and cell secretions were collected.

### Purification of activited protein from cell secretion

400 mL of cell secretion from 4 g of lamprey supraneural body was dialyzed in buffer A consisting of 20 mM KPB, 0.1 M KCl and 5% Glycerol, pH 7.0 at 4 °C. The dialyzed fraction was filtrated through a 0.22 μM membrane and then was applied to a 10 mL × 2 of Macro-Prep Ceramic Hydroxyapatite column equilibrated with buffer A. After the sample application, the column was washed with the same buffer and then eluted with a linear gradient from 0 to 250 mM KPB in buffer A. The pooled fractions containing protein activity from above column was dialyzed in buffer B consisting of 20 mM Tris-HCl and 5% Glycerol, pH 8.0 at 4 °C. The dialyzed fraction was applied to a 20 mL of Q Sepharose Fast Flow column equilibrated with buffer B. After applied and washed, the sample was eluted with a linear gradient from 0 to 300 mM KCl in buffer B. The fractions containing proteins with  activity were pooled. The preparation obtained 0.037 mg of active protein and was homogeneous when analyzed by SDS-PAGE. The active protein recovery was 69%.

### Mass spectrometry and protein identification

The purity and molecular mass of the protein were determined by 12% SDS-PAGE under reduced conditions. Proteins were visualized with 0.25% Coomassie Brilliant Blue R-250 in 50% methanol containing 10% acetic acid. In-gel tryptic digestion was done according to  the manufacturer’s protocol. The LC/MS/MS of tryptic peptides was performed on MALDI-TOF mass spectrometry using a Bruker Ultraflex mass spectrometer. The PMF-lift profiles obtained were used to search for peptide matches, using *ProFound*, run locally against our ESTs database of cDNA library from lamprey supraneural body tissues.

### Cloning, expression, and purification of the lamprey LIP

The open reading frame (ORF) of the LIP gene, which was flanked by a *Bam*HI and a *Hind*III restriction site, was amplified and subcloned into a pCold I vector with a His-tag. The recombinant LIP was expressed in *RosettaBlue* competent cells induced with 1 mM isopropyl-1-thio-β-D-galactopyranoside (IPTG) for 12 h at 16 °C. LIP purification was performed by Ni-NTA His-Bind resin column.

### Production of an anti-LIP monoclonal antibody and western blotting

The anti-LIP monoclonal antibody, which was produced using recombinant LIP as an antigen to immunize Balb/c mice, was kindly provided by GenScript Corporation (NanJing, China). For the western blots, 1 μg of purified recombinant LIP (rLIP) and native LIP (nLIP) was subjected to 12% SDS-PAGE and transferred onto nitrocellulose membranes. The membranes were blocked with 5% skim milk and incubated with mouse anti-LIP (1:1000 dilution) antibody overnight at 4 °C, followed by incubation with HRP-conjugated goat anti-mouse IgG (1:5000). The membrane was developed with the ECL substrate (Beyotime, China).

### Live-cell imaging and 3D-SIM super-resolution microscopy

Cells were plated onto confocal dishes (coverglass-bottom dishes) and treated with LIP at 37 °C. A time series of live-cell images was obtained using a Zeiss LSM 780 inverted microscope (Carl Zeiss, Inc.) and analyzed using Zeiss ZEN LE software.

Cells were plated onto confocal dishes (coverglass-bottom dishes) and stained with Hoechst (Sigma) for 20 min to visualize the cell nuclei. Then, the cells were washed twice with phosphate-buffered saline (PBS) and stained with CellMask™ Plasma Membrane Stains/MitoTracker Mitochondrion-Selective Probes/ER tracker™ Dyes (Molecular Probes, Invitrogen Corporation, Carlsbad, CA) for 4 min/30 min/30 min. The samples were labeled with Alexa488-tagged LIP or LIP after washing with PBS buffer and analyzed on a Zeiss LSM 780 inverted microscope (Carl Zeiss, Inc.) or by 3D-SIM super-resolution microscopy. The 3D-SIM images of cells were acquired on a DeltaVision OMX V4 imaging system (GE Healthcare).

### Cytosolic calcium ion concentration determination

To detect intracellular Ca^2+^ levels, the Ca^2+^-specific fluorescent dye Fluo3-AM was loaded into MCF-7 cells treated with 0.5 μg/mL LIP using a modified procedure adapted from the manufacturer (Beyotime, China). Briefly, cells were incubated for 12 h at 37 °C with 4 mM Fluo3-AM in Hank’s buffered salt solution (HBSS) with or without 1.3 mM Ca^2+^. Then, the cells were washed two times with fresh HBSS and incubated in HBSS at room temperature. The Fluo3-AM green fluorescence (Excitation: 488 nm; Emission: 526 nm) was proportional to the intracellular Ca^2+^ concentration. The changes in Fluo3-AM intensity in response to LIP exposure were monitored using a BD Biosciences FACSCanto flow cytometer.

### Protein efflux measurements

Cells were plated in 6-well plates at a density of 2 × 10^6^ cells/well and cultured overnight. After two washes with PBS, the MCF-7 cells were incubated with 0.5 μg/ml LIP for 12 h at 37 °C. Cell pellets and supernatants were independently collected, and the leakage of various proteins located in the cytosol and organelles into the medium and cell fractions were separated by SDS-PAGE and detected by western blotting using appropriate antibodies.

### Real-time PCR and western blot analysis

The cells were treated with 0.5 μg/ml LIP for 0 h and 24 h. RNA was isolated and purified using the RNeasy Mini Kit (Qiagen), using 10 ng RNA per reaction. Targets were amplified with the OneStep RT-PCR Kit (Qiagen) using oligo(dT)12 as a primer. Each sample was run in triplicate. The relative RNA amounts were calculated with the ΔΔCt method and normalized to an internal control, glyceraldehydes-3-phosphate dehydrogenase (GAPDH). TNF-α: 5′- TGTAGCCCATGTTGTAGCAAACC -3′ (forward) and 5′-GAGGACCTGGGAGTAGATGAGGTA-3′ (reverse); IL-1β: 5′- CTGAGCACCTTC TTTCCCTTCA-3′ (forward) and 5′-TGGACCAGACATCACCAAGCT-3′ (reverse).

The cells were treated with 0.5 μg/ml LIP for 0, 12 and 24 h. The cells were lysed in lysis buffer (20 mM Tris-HCl, pH 7.4, 1% Triton X-100, 10% glycerol, 150 mM NaCl, 1 mM phenylmethanesulfonyl fluoride (PMSF), and 1 μg/mL each leupeptin, antipain, chymostatin, and pepstatin A) on ice for 1 h and centrifuged (9500×g, 15 min). Equivalent amounts of total proteins were separated by SDS-PAGE and transferred to nitrocellulose membrane, and the presence of various proteins was monitored by immunoblot analysis following standard procedures. The following antibodies were used: mouse RIPK1 monoclonal antibody (Abnova), mouse RIP3 monoclonal antibody (2 μg/mL, ProSci), rabbit caspase 1 polyclonal antibody (2 μg/mL, Proteintech), mouse anti-TNF-α monoclonal antibody (2 μg/mL, Abcam), mouse anti-IL-1β monoclonal antibody (2 μg/mL, Proteintech), rabbit anti-actin polyclonal antibody (1 μg/mL, Proteintech).

### LDH efflux and PI influx measurements

For determination of LDH efflux from the cells, the medium was centrifuged to remove floating cells. Next, the supernatant was mixed with the solution of the LDH cytotoxicity detection kit (Takara), and the optical densities at 490 nm were measured with microplate reader model 550(Bio-Rad). The amounts of leaked LDH were determined and represented as percentages of the LDH activity obtained after treatment of the cells with 1%(*w*/*v*) Triton X-100. For PI (sigma) staining, cells (5 × 10^5^ cell/sample) were collected by centrifugation and washed twice with PBS, before PI (final concentration:5 μg/ml) in 1640 media was added together with rLIP and nLIP. At the indicated times, the uptake of PI into the cells was measured with a FACScan flow cytometer (EPICS XL; Beckman Coulter, Fullerton, CA). Cell size was evaluated by forward-angle light scattering. PI-negative cells with a normal size were considered living.

### GeneChip microarray

Total RNA from each group (PBS-treated MCF-7 cells, LIP–treated MCF-7 cells, PBS-treated K562 cells and LIP–treated K562 cells) was analyzed with a GeneChip microarray (Agilent human gene expression 4 × 44,000 microarrays; Shanghai Biochip). The raw GeneChip microarray data were deposited in the Gene Expression Omnibus at http://www.shbiochip.bioon.com.cn. For principal -component analysis (PCA) and cluster analyses, the SBC Analysis System (http://sas.shbio.com/portal/root/molnet_shbh/index.jsp/) provided by Shanghai Biochip was used.

### Tumor xenograft assay

Seven-week-old female and male nude mice were used to establish xenografts. For each injection, 4 × 10^6^ cells were collected and re-suspended in 100 μL of ice-cold 20% matrigel (BD Bioscience) in PBS (Gibco). This 100 μL solution was injected subcutaneously into nude mice using a 22-gauge needle. Transplantations of MCF-7 cells were allowed to grow for 2 weeks, whereas xenografts with MCF-7 cells were divided into two groups according to the tumor volume. Two groups of animals received intratumorally injections of PBS or rLIP into the sites of tumors every 2 days. Tumors were examined twice weekly; length and width measurements were obtained with calipers, and tumor volumes were calculated using the equation (L × W^2^)/2. Human tumor xenografts in a nude mice model were allowed to grow for a month from injection. Animals were killed; tumors were excised, weighed, and paraffin embedded.

### Immunohistochemistry and H&E staining

Serial 4.0-μm sections were cut and subjected to immunohistochemical and H&E staining. After deparaffinization, sections were immunohistochemically analyzed using an anti-Ki67, anti-LIP, and anti-F4/80 antibodies (Abcam, Cambridge, MA) or H&E stained with Mayer’s hematoxylin solution. Apoptotic cells were confirmed with the In Situ Cell Apoptosis Detection Kit, AP (Sangon Biotech), in accordance with the manufacturer’s instructions. The images were captured using the ZEN Blue Lite microscope software (Zeiss Scope. A1, Germany).

### Preparation of artificial membrane liposomes containing calcein

Artificial membrane liposomes were prepared as previously reported [4]. A dry thin membrane of a mixture of phosphatidylcholine (PC) and cholesterol (CHL) or of a mixture of phosphatidylcholine (PC) and phosphatidylserine (PS) was prepared by evaporating a solution of the lipid and the polymer in methanol and by subsequent drying under a vacuum. The membrane was dispersed in aqueous 200 mM calcein solution (pH 7.4) and sonicated for 30 min using an ultrasonic disruptor (Tomy Seiko, UD-200). Free calcein was removed by gel chromatography on a Sephadex G-50 (Pharmacia, Sweden) column using buffer (pH 7.4) consisting of 50 mM Tris-HCl and 100 mM NaCl. Liposomes containing calcein were prepared according to the above procedure. The precise artificial membrane liposome concentration was determined using an inductively coupled plasma-optical emission spectrometer.

### Calcein leakage experiments

To determine the effect of LIP on the leakage of the artificial membrane liposomes, liposomes and 1 μg LIP (or control protein, 10% Triton X-100) were added to black 96-well microtiter plates and fluorescence values were measured at different times using a Thermo Scientific Varioskan Flash (Thermo scientific, USA). Our standard curve describing the fluorescence intensity as a function of calcein concentration showed that the fluorescence intensity of calcein was directly proportional to the reaction time. The reaction buffer led to self-quenching of its fluorescence, resulting in low background fluorescence intensity of the vesicle dispersion (Fo). The release of calcein, which was caused by LIP addition, led to the dilution of the dye into the medium and, therefore, could be monitored by the increase in fluorescence intensity (Ft). The experiments were normalized relative to the total fluorescence intensity (Fmax), which corresponded to the total release after complete disruption of all the vesicles by Triton X-100 (0.1%). The results of the leakage experiments are presented as the percentage of released calcein, which was calculated as follows: RF(%) = 100(Ft-F0)/(Fmax-F0). The calcein fluorescence excitation and emission wavelengths were 470 nm and 520 nm, respectively.

### Isothermal titration calorimetry experiments

ITC experiments were performed on a MicroCal ITC-200 system (GE Healthcare) at 25 °C in PBS buffer composed of 137 mM NaCl, 2.7 mM KCl, 14.1 mM Na_2_HPO_4_·12H_2_O, and 1.5 mM KH_2_PO_4_, pH 7.2. LIP (10 μM) was purified and dialyzed against the PBS buffer, and the pH values were adjusted to match that of the protein solution. PS (100 μM) was dissolved in the PBS buffer, centrifuged, and degassed before the experiment. In a typical binding experiment to characterize the PS/PROTEIN interaction, which was performed using the ITC200 system, 50 μM PS in a final volume of 200 μL was injected into a sample cell, and 10 μM LIP was injected by syringe into the reaction chamber in 2 μL pulses at 5 min intervals. These data were processed using MicroCal Origin 5.0 software.

### Statistical analysis

Statistical analysis was performed in GraphPad Prism. All data were obtained from independent experiments. Error bars in the figures represent the standard deviation (SD). n values are specified in the figure legends.

## Results

### Purification and identification of native active protein

**Fig. 1:**
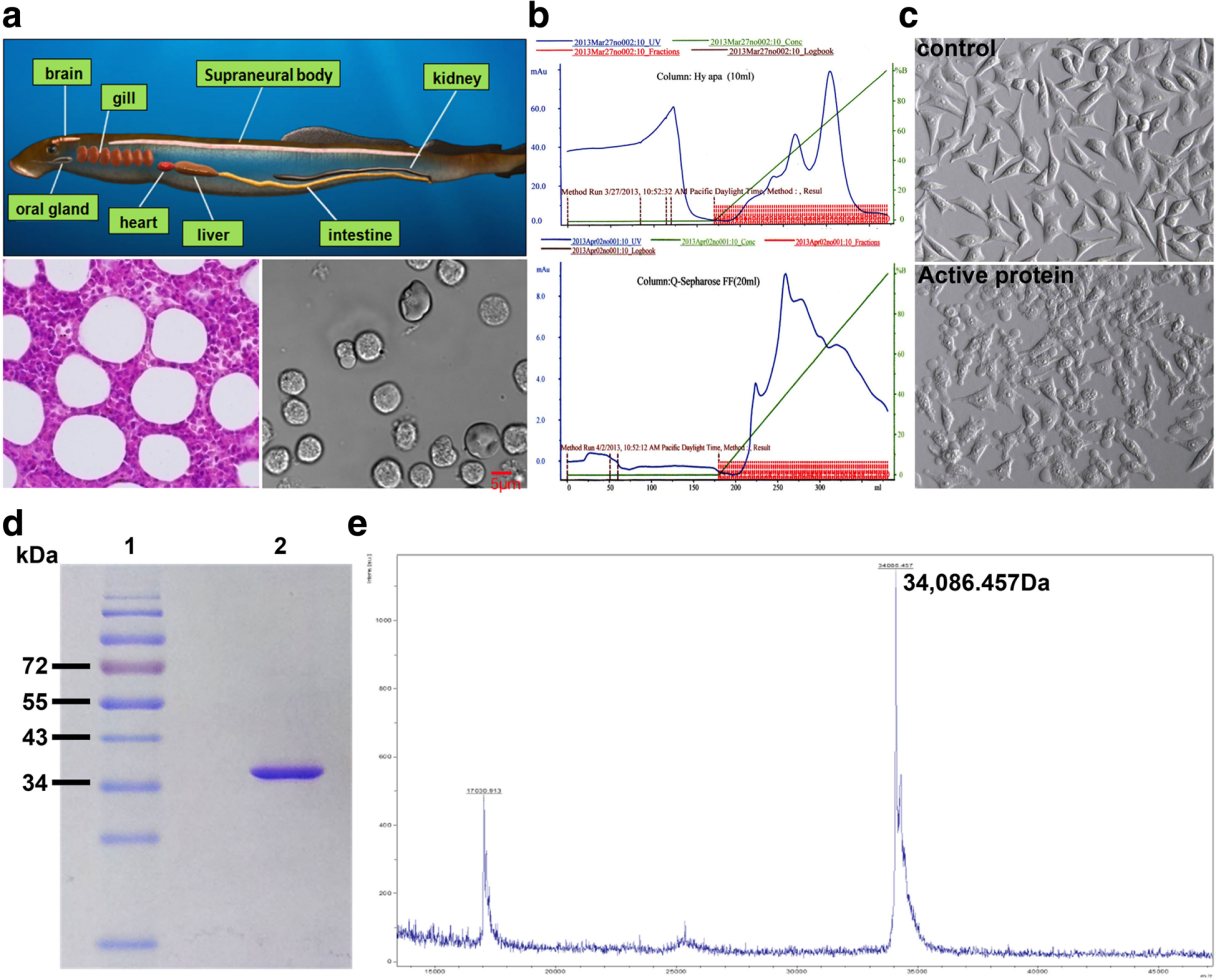
Identification of active protein from supraneural body in the lamprey. **a** Tissue of the supraneural body by hematoxylin and eosin (left pane). Types of cells from the supraneural body (right pane). **b** Macro-Prep Ceramic Hydroxyapatite column of flow-through and eluted fraction. – green line, KPB gradient; − blue line, absorbance at 280 nm; □ activity protein(upper pane). Q Sepharose Fast Flow column of flow-through and eluted fraction. – green line, KCl gradient; − blue line, absorbance at 280 nm;□ activity protein (low pane). **c** MCF-7 cells were seeded at 5 × 10^4^ cells in a 96-well plate. Each of the purified fractions were added to MCF-7 cells, and photographs were taken after the 30 min using optical microscope. (Magnification: 100×). **d** Determination of the purity and molecular mass of LIP via SDS-PAGE under reduced conditions. Lane 1, protein molecular markers; lane 2, native active LIP. **e** Identification of the molecular weight of LIP through MALDI-TOF mass spectroscopy

In the feeding adult, the supraneural body was well developed, extending from the third gill pouch to the anterior part of the second dorsal fin. The tissue contain a variety of blood cells between the adipose cells (Fig. 1a). Earlier studies on lampreys suggested that cell secretions from supraneural body tissues possesses cytocidal activity against tumor cells [5]. To identify the protein involved in this activity, cell secretions were purified using a hydroxyapatite column and a Q Sepharose Fast Flow column (Fig. 1b). The fraction of protein activity is determined by the degree of disruption of the cell membrane (Fig. 1c), When these fractions with active protein were collected and analyzed via 12% SDS-PAGE, a protein band was observed at approximately 34 kDa molecular weight (Fig. 1d). According to MALDI-TOF mass spectroscopy, the molecular mass of the purified protein was determined to the 34,086.457 Da (Fig. 1e).

### Peptide mass fingerprinting and de novo sequences of the active protein

**Fig. 2:**
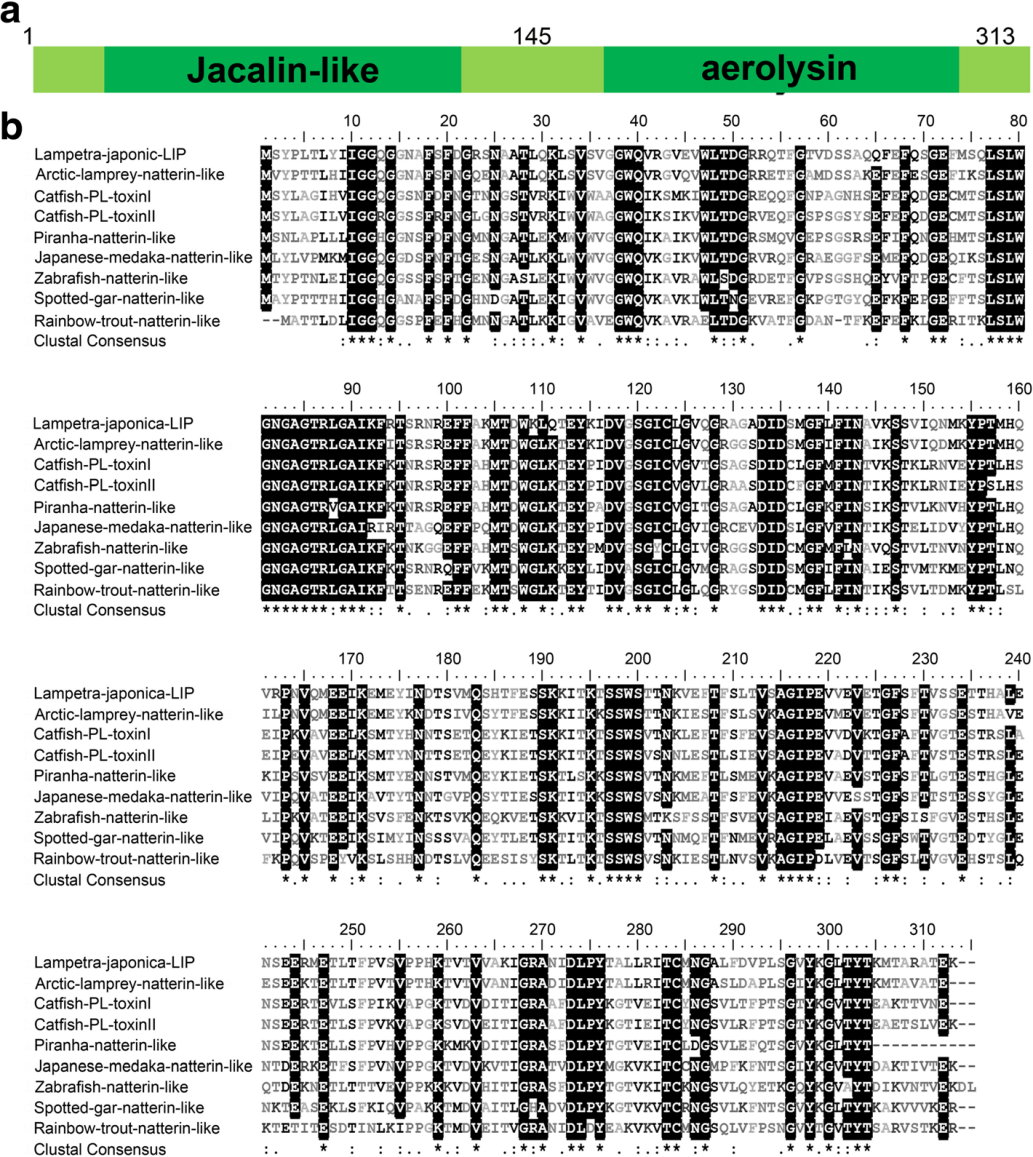
Sequences analysis and the predicted structure of the LIP. **a** LIP have the N-terminal β-prism lectin module and the C-terminal aerolysin module, respectively. **b** Sequence alignment analysis

The LC/MS/MS analysis of tryptic-digested peptides of the purified novel protein identified 10 unique peptides with greater than 95% probability(Table 1). When these de novo peptide sequences were subjected to a BLAST search from a cDNA library of SB cells in the lamprey against a protein database, these sequences showed significant similarity (score 556.59, rank 1, 26.20% sequence coverage), termed LIP. The nucleic acid sequence of LIP was detected in a cDNA library of lamprey supraneural body tissues. A 942-bp lamprey LIP gene fragment was identified, encoding a 313-aa protein (GenBank:MF627695) with an N-terminal jacalin-like domain and a C-terminal aerolysin domain (Fig. 2a). Indeed, the aerolysin domain-containing proteins are widely distributed, from bacteria to vertebrates. Specifically, the aerolysin domain of LIP showed high similarity to the aerolysin domains of other vertebrate proteins. For comparison, the amino acid sequences of the fish natterin-like proteins are also shown in Fig. 2b. Moreover, amino acid sequence identities among lamprey and the fish natterin-like proteins are summarized in Table 2. LIP share as high as 83% sequence identity with natterin-like from *Lethenteron camtschaticum* and are also homologous (50–60% identities) with the fish natterin-like proteins. LIP sequence had higher homology with natterin-like gene, but LIP as an active protein played an important role in lampreys immune defense. However, presently, there has been no evidence that natterin-like protein from lamprey has biological activity. Together, we named the novel active protein with cytocidal actions against tumor cells as lamprey immune protein (LIP).

**Table 1:** LC/MS/MS analysis of tryptic-digested peptides of LIP

m/z meas	Score	Sequence
416.7327	19.8	R.SNAATLQK.L
594.3200	85.8	K.LSVSVGGWQVR.G
566.2860	62.7	R.GVEVWLTDGR.R
402.7601	16.9	R.LGAIKFR.T
456.2394	24.1	R.NREFFAK.M
884.9258	71.6	R.AGADIDSMGFLFINAVK.S
453.7385	35.4	K.SSVIQNMK.Y
791.8974	63.4	R.METLTFPVSVPPHK.T
680.3705	53.9	R.ANIDLPYTALLR.I
1001.4636	53.1	R.ITCMNGALFDVPLSGVYK.G

**Table 2:** The LIP sequence identity of *L. japonica* compared with homologs of other species

Species (common name)	Gene	NCBI no	Sequence identity (%)
Arctic lamprey	Natterin-like protein	AFX60113.1	83%
Striped eel catfish	PL-toxin I	BAK19070.1	59%
Striped eel catfish	PL-toxin II	BAK19071.1	58%
Red-bellied piranha	Natterin-like protein	XP_017560326	60%
Japanese medaka	Natterin-like protein	XP_004086107.1	60%
Zebrafish	Natterin-like protein	NP 001013322.1	55%
Spotted-gar	Natterin-like protein	xp-006630213.1	59%
Rainbow trout	Natterin-like protein	Xp-021422815.1	55%

### Cytocidal activity of recombinant LIP against human tumor cells

**Fig. 3:**
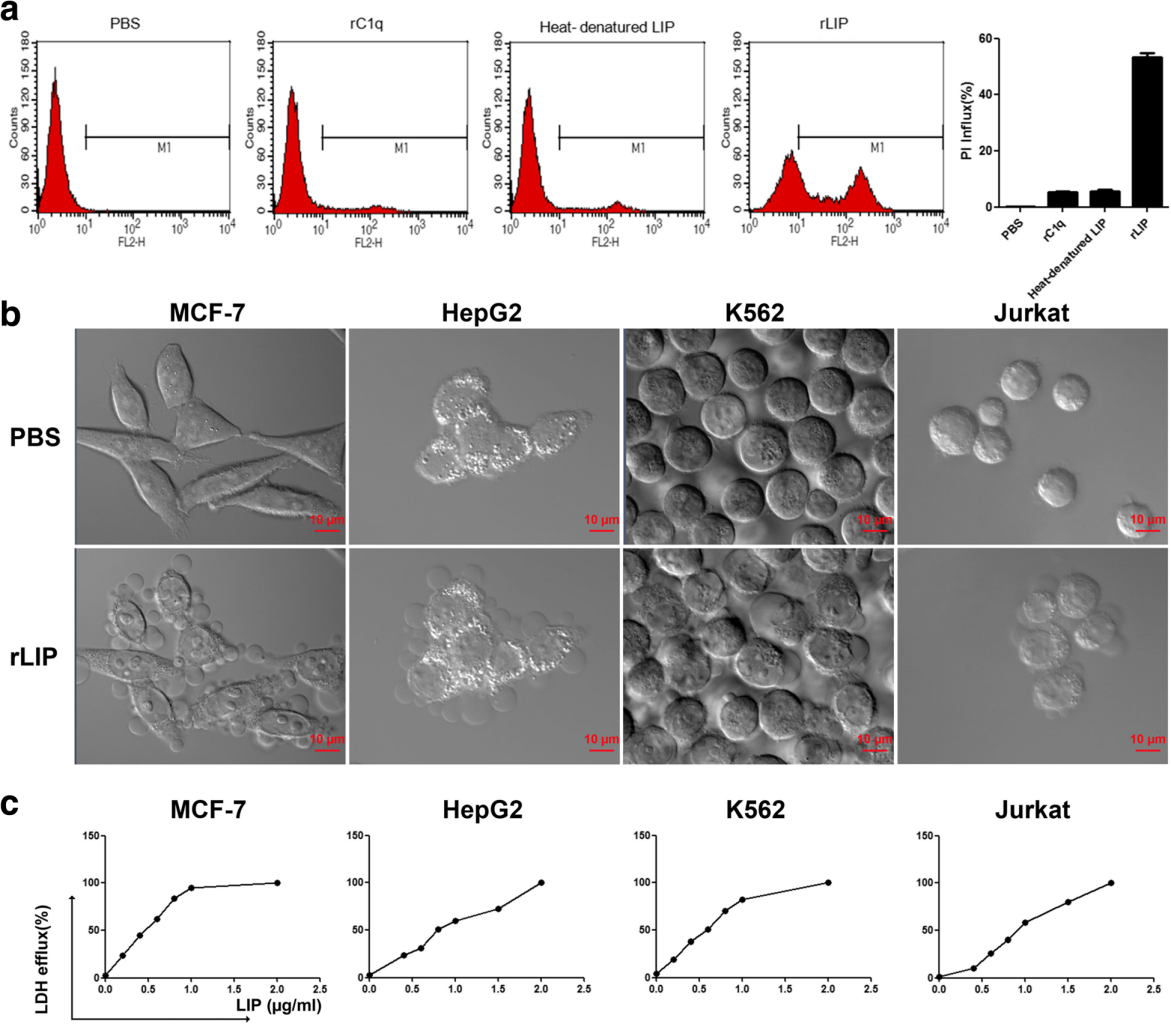
Cytocidal activity of recombinant LIP. **a** MCF-7 cells were incubated with unrelated protein purified from *E.coli* (rC1q), heat-denatured LIP (0.5 μg/mL) and recombinant LIP (0.5 μg/mL) for 12 h at 37 °C. Cell death was analyzed by PI staining and by flow cytometry. PBS-treatment cells were used as a negative control (left pane). Histogram showing statistics of the above results (right pane). All experiments were repeated at least three times with similar results. **b** The cells were incubated with 0.5 μg/mL LIP at 37 °C and visualized by Live-Cell Imaging. **c** Cytocidal activity of LIP against cultured tumor cells. A total of 2 × 10^4^ cells were preincubated at 37 °C for 20 h and then treated with LIP (final concentrations, 0.2 μg to 2 μg) for 12 h at 37 °C. Cytocidal activity of LIP was determined using the LDH cytotoxicity assay kit

The full-length cDNA of LIP was cloned by PCR. The purified PCR product was digested with *Bam*HI/*Hind*III and cloned into the pCold I vector (4407 bp), which was treated with the corresponding enzymes (Additional file 1: Figure S1). Recombinant LIP was expressed as a histidine-tagged fusion protein in Rosetta Blue competent cells. The purified rLIP migrated as a single band on a 12% SDS-PAGE gel, with a molecular mass of approximately 35 kDa (Additional file 1: Figure S1). The BCA assay demonstrated that the concentration of purified recombinant LIP (rLIP) was approximately 1 mg/mL. The level of cell death induced by rLIP-treatment was close to that observed after the same dose of native LIP-treatment (Additional file 2: Figure S2). To ensure that the death induced by LIP is not simply due to toxic contaminating *E.coli* proteins, heat-denatured LIP and unrelated protein purified from *E.coli* were included as specificity controls (Fig. 3a). LIP markedly induced tumor cell death, which was characterized by balloon-like shapes and, finally, by cell disintegration (Fig. 3b, Additional file 3: Video S1a and Additional file 4: Video S1b). Dose responses of LIP on the viability of various cultured human tumor cells were monitored using the LDH assay, and the LD50 values 12 h after LIP administration are shown in Fig. 3c and Table 3. A sufficient amount of LIP protein could be purified from *E.coli*, allowing us to explore its physiological and pathological functions without genetic manipulation, which is very hard to perform in Lamprey.

**Table 3:** Cytocidal activities of LIP against various tumor cells

Cell name	Characteristics	LD50(μg/ml)
MCF-7	Breast adenocarcinoma cell	0.52
HepG2	Hepatocyte cancer	0.75
K562	Chronic myeloid leukemia	0.63
Jurkat	Leukemia T cells	0.98


Additional file 3: Video S1a. LIP induced cell death in MCF-7 cells. (1040 kb)



Additional file 4: Video S1b.LIP induced cell death in K562 cells. (1740 kb)


### LIP increases plasma, mitochondrial and endoplasmic reticulum (ER) membrane permeability in cells

**Fig. 4:**
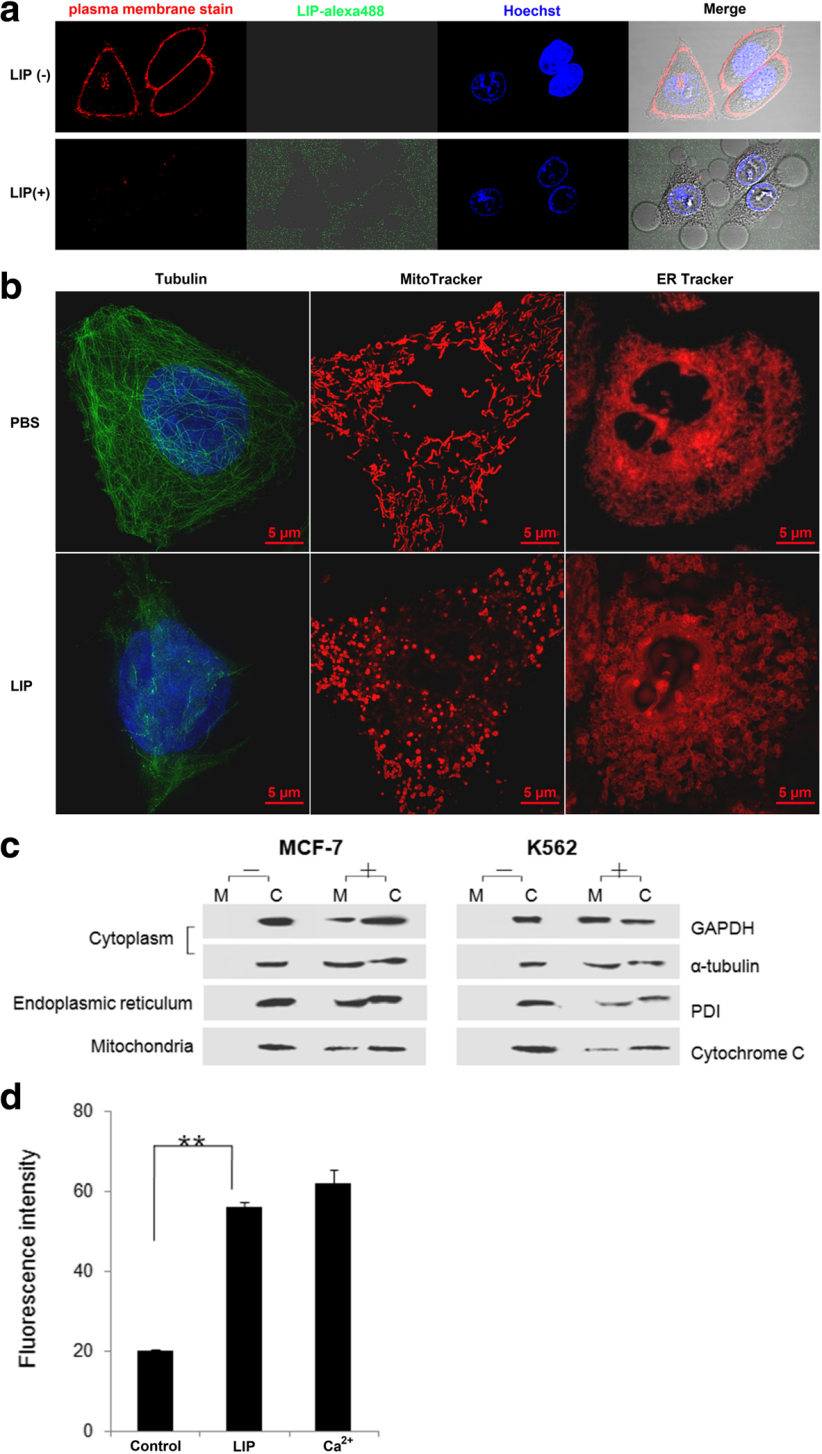
Morphological changes in cells upon exposure to LIP. **a** Live-cell fluorescence images of MCF-7 cells treated with FITC-tagged LIP. MCF-7 cells were incubated in medium containing 0.5 μg CellMask™ orange plasma membrane stain for 4 min and then incubated with FITC-tagged LIP (1.0 μg/ml). The cells were observed using an Olympus FluoView FV1000 confocal microscope and photographed at the indicated time points. (Magnification: 63×). **b** Microtubule and mitochondrial fragmentation and ER vacuolation. MCF-7 cells were incubated with or without LIP (0.5 μg/mL) at 37 °C for 12 h. The cells were stained with a monoclonal antibody against tubulin and incubated in medium containing 30 nM MitoTracker Red or ER tracker. Merged images of cells double-stained with DAPI are shown. **c** Leakage of various proteins from the cytosol and organelles. MCF-7 cells were incubated with (+) or without (−) 0.5 μg/mL LIP at 37 °C for 12 h. The culture medium and cells were independently collected, and four marker proteins in each fraction were separated by SDS-PAGE and detected by western blotting using appropriate antibodies. M and C indicate the medium and cell fractions, respectively. **d** Elevation of intracellular calcium concentrations in MCF-7 cells following LIP treatment. Each bar represents the mean value from three determinations with the standard deviation (SD). Data (mean ± SD) with asterisks significantly differ (**P < 0.01) between treatments

MCF-7 cells were double stained with orange plasma membrane stain and Hoechst 33258 nucleic acid stain. When the cells were incubated with Alexa488-labeled LIP, the results showed that LIP was primarily located on the plasma membrane (Fig. 4a). Whereas the reticular morphology of the microtubules and mitochondria was clearly visible by fluorescence in untreated control cells, the intracellular tubular network of the ER was converted into vacuolar structures. Furthermore, many vacuolar structures were observed in the cytoplasmic space during the early stage of LIP action, and the cytosolic volume was decreased, with deformation at the late stage. In addition, extensive cytoskeletal and mitochondrial fragmentation was observed in LIP-treated cells (Fig. 4b). To elucidate whether endomembrane systems and organelle morphologies were disrupted by the actions of LIP, the intracellular locations of proteins found in the effusions of LIP-treated cells were examined. We found that the cytoplasmic protein glyceraldehyde-3-phosphate dehydrogenase (GAPDH), α-tubulin, cytochrome C from the mitochondrial intermembrane space and protein disulfide isomerase (PDI) from the ER lumen were present in the medium but remained abundant in the cells (Fig. 4c). These results indicate that the plasma, mitochondrial and ER membranes are damaged by LIP. Importantly, Ca^2+^ was released by the actions of LIP (Fig. 4d), indicating that the altered morphology correlated with plasma membrane damage. These observations demonstrate that LIP disrupts membrane systems and causes alterations to organelle morphology, with release of the plasma, mitochondrial and ER proteins.

### LIP-induced expression of genes encoding inflammatory molecules

**Fig. 5:**
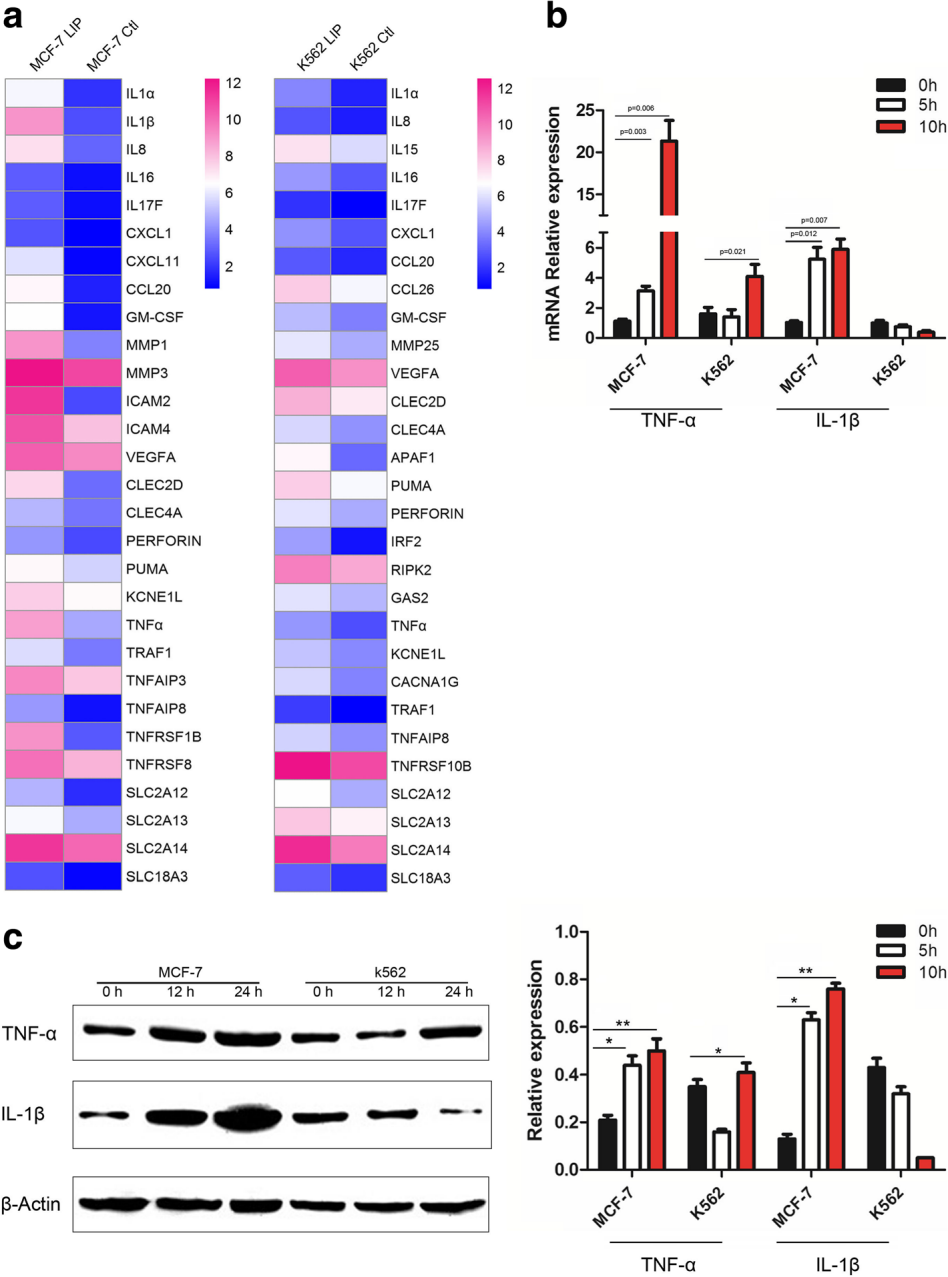
LIP can significantly increase the expression of inflammatory molecules in MCF-7 cells. **a** Heat map representation of candidate genes involved in the pathways induced by LIP. Blue and red colors represent low-to-high expression levels, and the color scales correspond to the expression values of the microarray. **b** Q**-**PCR analysis of inflammatory molecule (TNF-α, IL-1β) expression in MCF-7 and K562 cells incubated with LIP for different times. Total RNA was quantified by qRT-PCR and normalized to gapdh expression. **c** Western blot analysis of inflammatory factor expression in MCF-7 and K562 cells. Western blot analysis for the expression of TNF-α & IL-1β in MCF-7 and K562 cells incubated with LIP for different times. β-actin served as a loading control(left pane). Histogram showing statistics of the above results (right pane). Means ± SDs are shown (*n* = 3 per group). **P < 0.01

To detect the gene expression differences between cells with LIP-treatment and without LIP-treatment cells, gene expression profiling coupled with bioinformatic analyses was done. This revealed that a more than 2 fold change difference in mRNA transcript levels in MCF-7 and K562 cells treated with LIP-treatment than in the cells without LIP-treatment (Fig. 5a). The expression levels of inflammatory molecules were further analyzed via real-time PCR using total RNA obtained from untreated or LIP-treated tumor cells (Fig. 5b). The degree of gene expression changes determined through real-time PCR and microarray analyses showed agreement. Next, MCF-7 and K562 cells were treated with LIP for different times (0, 12, 24 h) and analyzed through western blotting with antibodies against TNF-α and IL-1β. The results further confirmed that treatment with LIP at all tested time points significantly increased TNF-α and IL-1β gene expression in MCF-7 cells, and TNF-α gene expression increased in K562 cells only at 24 h (Fig. 5c). Together, these data suggested that TNF-α and IL-1β production in response to LIP induced a cascade amplification reaction. Furthermore, pro-inflammatory marker production by LIP-induced enlarged cell death.

### The regulation of cell death by LIP is dependent on the pyroptosis or necroptosis pathway

**Fig. 6:**
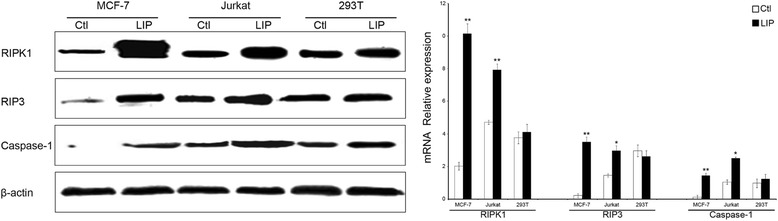
LIP induced pyroptosis or necroptosis pathway. Western blot analysis of RIPK1, RIP3 and Caspase-1 proteins expression using specific antibodies. β-actin was used as a loading control. Histogram showing statistics of the above results. Data are presented as the mean ± SEM of three independent experiments performed in duplicate. Three types of cell lines showed different increases in the levels of cellular proteins in response to LIP stimulation. *P < 0.05, **P < 0.01

Necrosis, which involves the swelling of organelles and plasma membrane rupture, was once considered accidental cell death caused by overwhelming physical or chemical trauma [6, 7]. However, we now know that specific genes can induce necrosis in a regulated manner. The receptor interacting protein kinase 3 (RIP3/RIPK3) has emerged as a critical regulator of programmed necrosis/necroptosis, an inflammatory form of cell death with important functions in pathogen-induced and sterile inflammation [8, 9]. Pyroptosis is a pro-inflammatory process that is dependent on caspase 1. Furthermore, pyroptosis leads to the release of intracellular contents, including HMGB1 and ATP, both of which can act as DAMPs (death associated molecular patterns) to stimulate further inflammation [10, 11]. To assess if the spontaneous cell death caused by LIP is dependent on the pyroptosis or necroptosis pathway, RIP1/3 kinase activity and caspase 1 activity were identified by western blotting. The results showed that RIPK1, RIP3 and caspase 1 proteins expression increased in the LIP-treatment group (Fig. 6a). Briefly, the spontaneous cell death was not only dependent on caspase 1 but also RIP1/3 kinase activity.

### LIP preferentially kills tumor cells in vivo

**Fig. 7:**
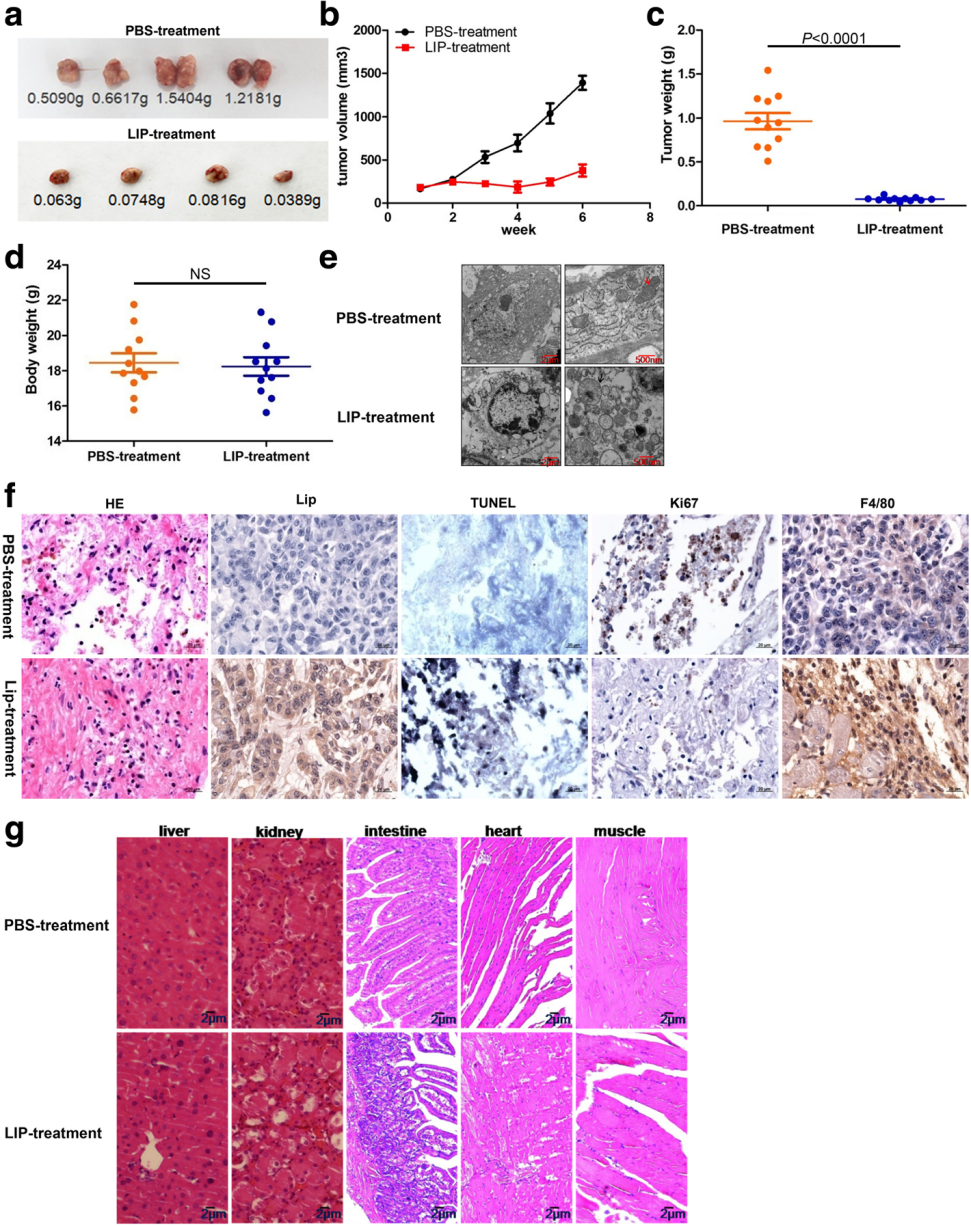
The effect of LIP treatment on cancer tissues and non-cancer tissues in vivo. **a** Mice with tumor growths were injected intratumorally (i.t.) 20 μg/kg rLIP or PBS. **b**-**d** Tumor growth of tumor-bearing nude mice were treated with either PBS or rLIP (*n* = 11 per group). Tumor size (**b**), tumor weight (**c**), body weight (**d**) of tumor-bearing nude mice. **e** Effect of LIP treatment on tumor tissues from xenografts. The cancer tissue pieces were treated with 2.5% glutaraldehyde for 24 h at 4 °C, and then a thin section of cancer tissues was observed via transmission electron microscopy (TEM). Red arrows, normal mitochondria; black arrows, mitochondria distension. **f** HE and IHC staining demonstrated that LIP induced the tumor cells death in vivo, as indicated by the expression of Ki67 and LIP, TUNEL-positive cells, and F4/80-positive cells. **g** Effect of LIP on non-cancer tissues from xenografts. Five different non-cancer tissues sections were stained by hematoxylin/eosin (H&E)

To evaluate the in vivo antitumor potential of LIP, we established preclinical human MCF-7 tumor models. First, the experimentally transformed human MCF-7 cell line yields vigorously growing tumor xenografts, which histopathologically resemble the invasive adenocarcinomas commonly encountered in breast cancer patients. Then, the mice with tumors received intratumoral injections of LIP into site-specific tumors every 2 days. As a control, mice with tumor growth received PBS injections, and showed that the tumors were larger and had higher tumor weights than the LIP-treated groups (Fig. 7a-c). Consistent with the in vitro experiments, evident antitumor effects were observed in LIP-treated animals. It is noteworthy that no obvious difference in body weight between control and LIP-treated groups was detected (Fig. 7d). In moderately differentiated cancer tissue, which has abundant cytoplasm with enlarged atypical nuclei and glandular structures, LIP injection caused a markedly degenerative appearance of the cells, and the plasma membrane of the cells was markedly perturbed compared to the untreated tumor cells, and the crista structure of mitochondria was disrupted (Fig. 7e). Immunohistochemistry analysis showed that tumors with PBS-treatment displayed increased Ki67-positive cells, and decreased TUNEL-positive apoptotic cells and F4/80-positive cells, whereas LIP-treated tumors showed decreased Ki67-positive cells, and increased TUNEL-positive apoptotic cells and F4/80-positive cells. In addition, injection of LIP into the tumor resulted in a vast amount of LIP localized on tumor cells, as well as the recruitment of macrophages, which directly induces tumor cells death (Fig. 7f). Slight morphological changes were observed in the endothelial cells of blood vessels, fibroblastic cells, and infiltrating inflammatory cells in the same tissue shown in Fig. 7g, even after LIP treatment. Taken together, these results indicate positive role of LIP in inducing tumor cell death in vivo.

### LIP induces exposure of phosphatidylserine and binds phosphatidylserine

**Fig. 8:**
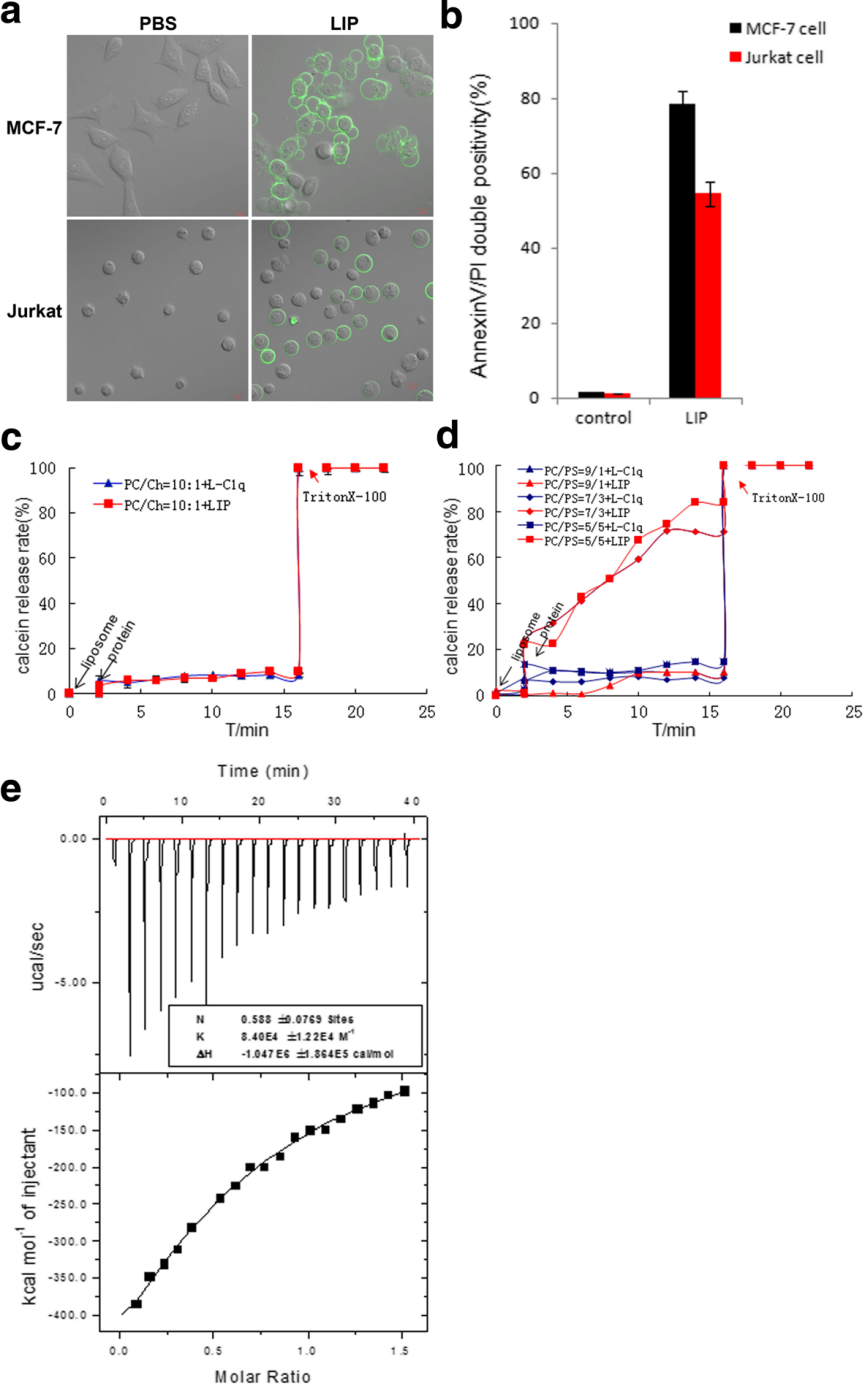
LIP induces exposure of phosphatidylserine and binds phosphatidylserine. **a** LIP induces exposure of phosphatidylserine. Cells were treated with LIP for 30 min. The cells were then imaged using Zeiss LSM 780 inverted microscope after staining for annexin-V-FITC. **b** LIP induces cell death in MCF-7 cells and Jurkat cells. Cells were treated with LIP for 12 h. The cells were then subjected to flow cytometric analysis after staining for annexin-V-FITC and propidium iodide, as mentioned in the methods. Shown are the data from three independent experiments. **c** Effect of LIP on a liposome membrane composed of a mixture of PC and CHL containing calcein. **d** Effect of LIP on a liposome membrane composed of a mixture of PC and PS containing calcein. **e** Calorimetric measurements of the LIP interaction with PS

Treatment with LIP led to the exposure of phosphatidylserine in MCF-7 cells and Jurkat cells as early as 12 h after treatment; the annexin-V single-positive cells were rare, while percentages of annexin-V/PI double positive cells were higher (Fig. 8a,b). To study the cytotoxic actions of LIP on the tumor cells, we simulated artificial membrane similar to mammalian cell membranes composed primarily of phosphatidylcholine (PC), cholesterol (CHL), and less phosphatidylserine (PS). Expression of phosphatidylserine was elevated in the outer membrane leaflet of human tumor cells [12–15]. A liposome membrane composed of a mixture of PC and CHL containing calcein fluorescence dye was incubated with LIP; the fluorescence intensity did not change with or without LIP treatment (Fig. 8c). However, a liposome membrane composed of a mixture of PC and PS containing calcein was incubated with LIP; an important feature of the fluorescence intensity curve is the fact that the curve reaches a plateau a few minutes after LIP addition. Even after a 10-min incubation, the fluorescence intensity did not significantly change once the plateau was reached. Furthermore, the curve indicated strong fluorescence intensity with the increase in PS content; recombinant L-C1q proteins used as a negative control in these experiments (Fig. 8d). In addition, isothermal titration calorimetry (ITC) was applied to analyze the relation between LIP and PS. As shown in Fig. 8e, LIP had an exothermic interaction with PS at 25 °C. The results revealed that the cytotoxic action of LIP depended on the PS content of the cell membrane. These results indicated that LIP initially induced exposure of phosphatidylserine, then bound phosphatidylserin, and finally enlarged the disruption of cell membrane structure.

## Discussion

In this study, we have identified the activation and cytocidal effects of LIP, which is a secretary protein produced by the SB tissues that preferentially kills human tumor cells. LIP induces remarkable alterations in cell morphology, such as cell swelling, blebbing, and subsequent lysis (Fig. 3b). After LIP treatment of MCF-7 cells and K562 cells, GAPDH, tubulin, cytochrome C and PDI protein efflux from the cells were observed to quickly occur (Fig. 4c), suggesting that LIP damages membrane permeability. In the present study, we also found that the intracellular tubular network of the ER was converted into vacuolar structures after LIP-treatment; LIP-treated tumor cells exhibited endogenous calcium leakage from the intracellular calcium stores (Fig. 4d), and in turn, this calcium leakage was able to significantly enhance the cytotoxicity. We speculated that LIP might be involved in the G protein-linked phosphatidylinositol signaling pathway, which then induced the intracellular Ca^2+^ leakage from the endoplasmic reticulum. Additional time and comprehensive studies are required to further investigate this possibility. In fact, the Cry1Ab toxin from an insecticidal *B. thuringiensis* strain participates in ion channel functioning in planar lipid bilayers [16]. The *Clostridium perfringens* toxin induces rapid changes in the cell membrane permeability to ions [17, 18].

Microtubules and microfilaments are polymer structures that orchestrate cellular movement, intracellular transport, cell division and signaling, involving interactions with a diverse range of proteins and signaling molecules [19]. After LIP treatment, we observed the presence of poorly cytoskeleton structure (Fig. 4b). In a previous study, cytochalasin B, which is a mitochondrial- and nucleic acid-directed drug, appears to preferentially damage malignant cells in patients with leukemia [20]. Microtubule-targeting agents are widely used for the treatment of cancer. Colchicines are inhibitors of microtubule polymerization [21], whereas BAL27862 binds to tubulin proteins and potently inhibits tubulin assembly and is a potent inhibitor of tumor cell growth [22]. Our results show that LIP addition to tumor cells can destroy microtubule structures. It is worthwhile to note that LIP is a potent, microtubule-destabilizing agent with obvious effects on microtubule organization. In addition, extensive mitochondrial fragmentation was observed in the tumor cells incubated with LIP, whereas abnormal endoplasmic reticulum structures were observed in LIP-treated tumor cells (Fig. 4b), indicating that the altered morphology correlated with the plasma membrane damage. These observations demonstrate that LIP impairs respiratory function and disrupts organelle morphology with the release of mitochondrial and ER proteins (Fig. 4c). Kitada et al. also have reported morphologic alterations induced by parasporin-2 on MOLT-4 cells, such as cytoskeletal alterations and mitochondrial and endoplasmic reticulum fragmentation [16]. However, what can be deduced from these changes is that the mechanism of action of LIP in human cancer cells may differ from other toxins. LIP most likely binds to molecules of the plasma membrane first and disrupts the membrane structure, thus inducing the leakage of ions, water and protein molecules through the plasma membrane at the early stage of its actions (Fig. 4a). During the LIP treatment of MCF-7 cells, LIP was located at the plasma membrane; however, when MCF-7 cell membranes were disrupted, LIP was observed in the cytoplasm. Thus, the final destination of LIP for killing the cells should be on the cell surface, where the membrane damage occurs.

Mechanistic analyses showed that caspase 1, RIPK1 and RIP3 expression was upregulated following LIP treatment. In fact, different toxic proteins have different means of inducing cell death [16–18]. To identify genes whose expression changed in MCF-7 cells treated with LIP, total RNA from each group (with or without LIP-treatment) was analyzed by GeneChip microarray (Agilent mouse gene expression 4 × 44,000 microarray, Shanghai Biotechnology Co., Ltd). Microarray analyses revealed that stimulating MCF-7 and K562 cells with LIP significantly increased the production of pro-inflammatory molecules and chemokines, and increased the expression of genes in the calcium signaling pathway, ROS signaling pathway, and nature killer cell-mediated cytotoxicity pathway (http://sas.shbio.com/portal/root/molnet_shbh/index.jsp). These results revealed the complexity of the molecular machinery that drives LIP-mediated cell death in tumor cells.

However, we found that LIP only recognized and had cytotoxic effects against tumor cells and confirmed that LIP recognized PS in the tumor cell membrane, as determined by ITC analysis. In addition, LIP had a more intense cytotoxic effect with an increase in the PS content (Fig. 8). Boersma et al. reported that tumor cells displayed higher levels of PS due to their higher rate of cell division and apoptosis [23]. PS could be an accurate marker for all tumor cells, as tumor and non-tumor cells can be distinguished based on the characteristic of the exposure or non-exposure of PS, respectively [24]. At present, PS has been used as a design target for anticancer drugs, such as an antimicrobial peptide, in which short peptides with a positive charge can combine with the negative charge of PS in tumor cell membranes [25, 26]. We expect that this unique anti-tumor protein and its putative PS receptor will allow great progress to be made in certain medical fields, such as in the diagnosis and control of tumor cells. However, interactions between LIP and other phospholipids were analyzed by ITC, and LIP did not recognize PCs and phosphatidylethanolamines (PEs) of tumor cell membranes (Additional file 5: Figure S3). Additionally, to determine whether LIP may recognize other molecular of cancer cells membrane, further studies are required.

## Conclusion

In summary, our results are the first to identify a novel LIP protein from lamprey supraneural body. LIP exhibits strong cytocidal activities against human tumor cells, with markedly divergent target cell specificities. Our results revealed that LIP binds to PS molecules at the surface of susceptible tumor cells and efficiently kills tumor cells from tumor nude mice, with no effect on normal cells. LIP disrupts membrane structures, induces alterations of the cytoskeletal structure, causes fragmentation of mitochondria and the ER, and increases cytosolic calcium levels. Further studies are required to identify the function of LIP and its related proteins in the lamprey. We expect that the unique anti-tumor effects of LIP will allow great progress to be made in certain medical fields, such as in the diagnosis and control of tumor cells.

## Additional files

Additional file 1: Figure S1. The construction and expression of pColdI-rLi protein plasmid. (a) The construction of pColdI-rLIP plasmid. M, DL10000 Marker; 1, pColdTMIDNA; 2, pColdTMIDNA (*Bam*H Ι/*Hind* III); 3, PCR products of rLIP; 4, rLi protein(BamH Ι/Hind III); 5, pColdI-rLIP; 6, Double digestion products of pColdI-rLIP. (b) Total protein was separated by 12% SDS-PAGE under reducing conditions and stained with Coomassie Brilliant Blue R-250. Lane M, low molecular weight protein maker; Lane 1, crude lysate pre-induction; Lane 2, crude lysate post-induction; Lane 3, purified LIP. (PDF 234 kb)

Additional file 2: Figure S2. The cytotoxicity of native LIP and recombinant LIP detected by flow cytometry. The MCF-7 cells were incubated with native LIP and recombinant LIP at 37 °C for 12 h. Cell death was analyzed by PI staining and by flow cytometry. Untreated cells were used as a negative control. All experiments were repeated at least three times with similar results. (PDF 904 kb)

Additional file 5: Figure S3. Calorimetric measurements of the LIP interaction with PE/PC. Calorimetric measurements of the LIP interaction with PE and PC. (PDF 398 kb)

## Abbreviations

LIP: Lamprey immune protein
PI: Propidium iodide
PS: Phosphatidylserine
RIP3: Receptor interacting protein 3
RIPK1: Receptor interacting protein kinase 1
SB: Supraneural body

## References

[1] Gadhamsetty S, Marée AF, Beltman JB, de Boer RJ (2014). A general functional response of cytotoxic T lymphocyte-mediated killing of target cells. Biophys J.

[2] Petranovic D, Pilcic G, Valkovic T, Sotosek Tokmadzic V, Laskarin G (2014). Perforin- and granulysin-mediated cytotoxicity and interleukin 15 play roles in neurocognitive impairment in patients with acute lymphoblastic leukaemia. Med Hypotheses.

[3] Wu F, Chen L, Liu X, Wang H, Su P, Han Y, Feng B, Qiao X, Zhao J, Ma N, Liu H, Zheng Z, Li Q (2013). Lamprey variable lymphocyte receptors mediate complement-dependent cytotoxicity. J Immunol.

[4] Apiratikul N, Penglong T, Suksen K, Svasti S, Chairoungdua A, Yingyongnarongkula B (2013). In vitro delivery of curcumin with cholesterol-based cationic liposomes. Bioorg Khim.

[5] Pang Y, Wang S, Ba W, Li Q (2015). Cell secretion from the adult lamprey supraneural body tissues possesses cytocidal activity against tumor cells. SpringerPlus.

[6] Festjens N, Vanden Berghe T, Vandenabeele P (2006). Necrosis, a well-orchestrated form of cell demise: signalling cascades, important mediators and concomitant immune response. Biochim Biophys Acta.

[7] Zhou Z, Han V, Han J (2012). New components of the necroptotic pathway. Protein Cell.

[8] Chen W, Wu J, Li L, Zhang Z, Ren J, Liang Y, Chen F, Yang C, Zhou Z, Su SS, Zheng X, Zhang Z, Zhong CQ, Wan H, Xiao M, Lin X, Feng XH, Han J (2015). Ppm1b negatively regulates necroptosis through dephosphorylating Rip3. Nat Cell Biol.

[9] Wang Q, Ju X, Zhou Y, Chen K (2015). Necroptotic cells release find-me signal and are engulfed without proinflammatory cytokine production. In Vitro Cell Dev Biol Anim.

[10] Brough D, Rothwell NJ (2007). Caspase-1-dependent processing of pro-interleukin-1beta is cytosolic and precedes cell death. J Cell Sci.

[11] Geng Y, Ma Q, Liu YN, Peng N, Yuan FF, Li XG, Li M, Wu YS, Li BL, Song WB, Zhu W, Xu WW, Fan J, Su L (2015). Heatstroke induces liver injury via IL-1β and HMGB1-induced pyroptosis. J Hepatol.

[12] Connor J, Bucana C, Fidler IJ, Schroit AJ (1989). Differentiation-dependent expression of phosphatidylserine in mammalian plasma membranes: quantitative assessment of outer-leaflet lipid by prothrombinase complex formation. Proc Natl Acad Sci U S A.

[13] Utsugi T, Schroit AJ, Connor J, Bucana CD, Fidler IJ (1991). Elevated expression of phosphatidylserine in the outer membrane leaflet of human tumor cells and recognition by activated human blood monocytes. Cancer Res.

[14] Dong HP, Holth A, Kleinberg L, Ruud MG, Elstrand MB, Tropé CG, Davidson B, Risberg B (2009). Evaluation of cell surface expression of phosphatidylserine in ovarian carcinoma effusions using the annexin-V/7-AAD assay: clinical relevance and comparison with other apoptosis parameters. Am J Clin Pathol.

[15] Riedl S, Rinner B, Asslaber M, Schaider H, Walzer S, Novak A, Lohner K, Zweytick D (2011). In search of a novel target-phosphatidylserine exposed by non-apoptotic tumor cells and metastases of malignancies with poor treatment efficacy. Biochim Biophys Acta.

[16] Kitada S, Abe Y, Shimada H, Kusaka Y, Matsuo Y, Katayama H, Okumura S, Akao T, Mizuki E, Kuge O, Sasaguri Y, Ohba M, Ito A (2006). Cytocidal actions of parasporin-2, an anti-tumor crystal toxin from bacillus thuringiensis. J Biol Chem.

[17] Wong RS, Mohamed SM, Nadarajah VD, Tengku IA (2010). Characterisation of the binding properties of bacillus Thuringiensis 18 toxin on Leukaemic cells. J Exp Clin Cancer Res.

[18] Kitada S, Abe Y, Maeda T, Shimada H (2009). Parasporin-2 requires GPI-anchored proteins for the efficient cytocidal action to human hepatoma cells. Toxicology.

[19] Fife CM, McCarroll JA, Kavallaris M (2014). Movers and shakers: cell cytoskeleton in cancer metastasis. Br J Pharmacol.

[20] Trendowski M, Yu G, Wong V, Acquafondata C, Christen T, Fondy TP (2014). The real deal: using cytochalasin B in sonodynamic therapy to preferentially damage leukemia cells. Anticancer Res.

[21] Parthasarathy PT, Cho Y, Lockey R, Kolliputi N (2014). An old molecule with a new role: microtubules in Inflammasome regulation. Cell Biochem Biophys.

[22] Prota AE, Danel F, Bachmann F, Bargsten K, Buey RM, Pohlmann J, Reinelt S, Lane H, Steinmetz MO (2014). The novel microtubule-destabilizing drug BAL27862 binds to the colchicine site of tubulin with distinct effects on microtubule organization. J Mol Biol.

[23] Boersma HH, Kietselaer BL, Stolk LM, Bennaghmouch A, Hofstra L, Narula J, Heidendal GA, Reutelingsperger CP (2005). Past, present, and future of annexin A5: from protein discovery to clinical applications. J Nucl Med.

[24] Sharma B, Kanwar SS. Phosphatidylserine: A cancer cell targeting biomarker. Semin Cancer Biol. 2017. 10.1016/j.semcancer.2017.08.012 28870843

[25] Riedl S, Leber R, Rinner B, Schaider H, Lohner K, Zweytick D (2015). Human lactoferricin derived di-peptides deploying loop structures induce apoptosis specificallyin cancer cellsthrough targeting membranous phosphatidylserine. Biochim Biophys Acta.

[26] Blondelle SE, Lohner K (2010). Optimization and high-throughput screening of antimicrobial peptides. Curr Pharm Des.

