# Correction to: A novel protein derived from lamprey supraneural body tissue with efficient cytocidal actions against tumor cells

**DOI:** 10.1186/s12964-017-0202-1

**Published:** 2017-11-27

**Authors:** Yue Pang, Changzhi Li, Shiyue Wang, Wei Ba, Tao Yu, Guangying Pei, Dan Bi, Hongfang Liang, Xiong Pan, Ting Zhu, Meng Gou, Yinglun Han, Qingwei Li

**Affiliations:** 1 College of Life Science, Liaoning Normal University, Dalian, 116081 China; 2 Lamprey Research Center, Liaoning Normal University, Dalian, 116081 China

## Correction

Unfortunately, following publication of this article [1], it was noticed that the key in Fig. 5c incorrectly showed ‘0 h’, ‘5 h’ and ‘10 h’. The corrected version, showing ‘0 h’, ‘12 h’ and ‘24 h’, can be seen below and the original article has been updated to reflect this.

**Fig. 5:**
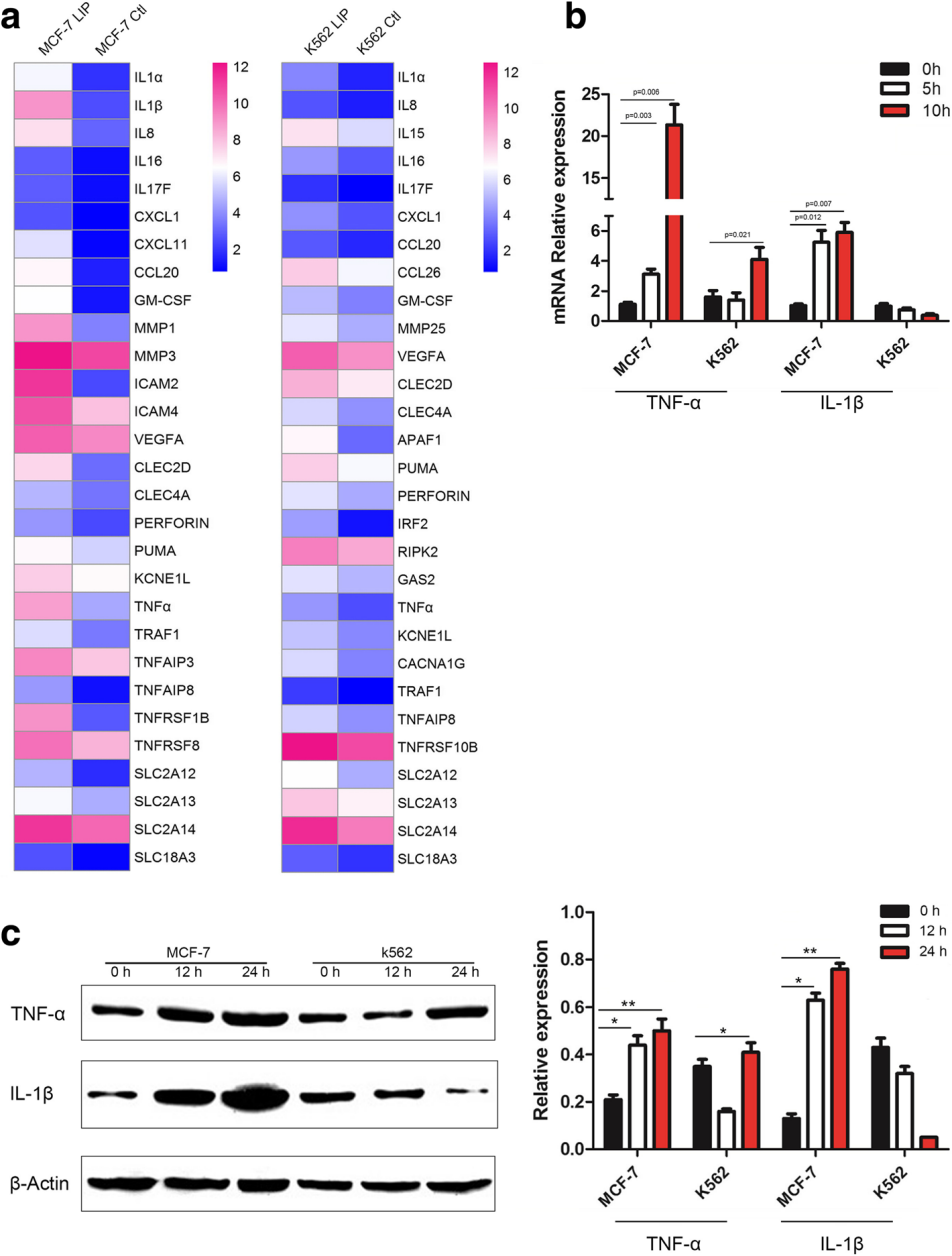
LIP can significantly increase the expression of inflammatory molecules in MCF-7 cells. **a** Heat map representation of candidate genes involved in the pathways induced by LIP. Blue and red colors represent low-to-high expression levels, and the color scales correspond to the expression values of the microarray. **b** Q-PCR analysis of inflammatory molecule (TNF-α, IL-1β) expression in MCF-7 and K562 cells incubated with LIP for different times. Total RNA was quantified by qRT-PCR and normalized to gapdh expression. **c** Western blot analysis of inflammatory factor expression in MCF-7 and K562 cells. Western blot analysis for the expression of TNF-α & IL-1β in MCF-7 and K562 cells incubated with LIP for different times. β-actin served as a loading control(left pane). Histogram showing statistics of the above results (right pane). Means ± SDs are shown (*n* = 3 per group). **P < 0.01
