# Supplementary figures and images for: A novel protein derived from lamprey supraneural body tissue with efficient cytocidal actions against tumor cells
Source: Cell Commun Signal. 2017 Oct 16;15:42. doi: 10.1186/s12964-017-0198-6 (PMC5644163; doi:10.1186/s12964-017-0198-6)

# Supplement Figure 1 Y Pang

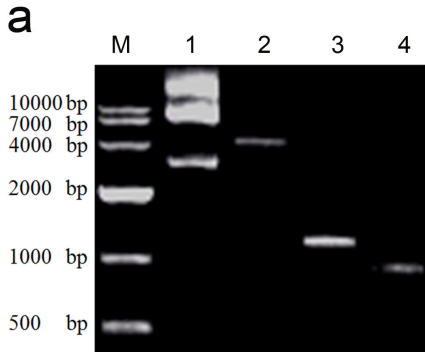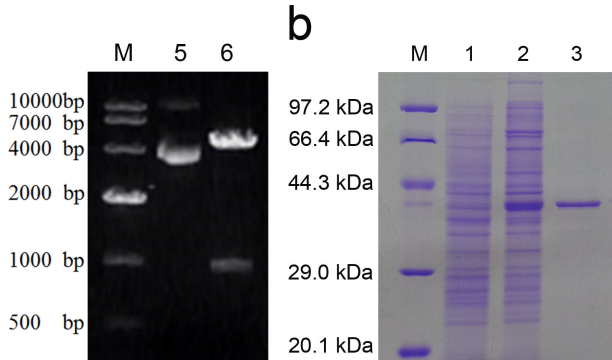

Supplement: Supplementary file 3 — The construction and expression of pColdI-rLi protein plasmid. (a) The construction of pColdI-rLIP plasmid. M, DL10000 Marker; 1, pColdTMIDNA; 2, pColdTMIDNA (BamH Ι/Hind III); 3, PCR products of rLIP; 4, rLi protein(BamH Ι/Hind III); 5, pColdI-rLIP; 6, Double digestion products of pColdI-rLIP. (b) Total protein was separated by 12% SDS-PAGE under reducing conditions and stained with Coomassie Brilliant Blue R-250. Lane M, low molecular weight protein maker; Lane 1, crude lysate pre-induction; Lane 2, crude lysate post-induction; Lane 3, purified LIP. (PDF 234 kb) [file 12964_2017_198_MOESM1_ESM.pdf]

## Supplement Figure 2 Y Pang

PBS control

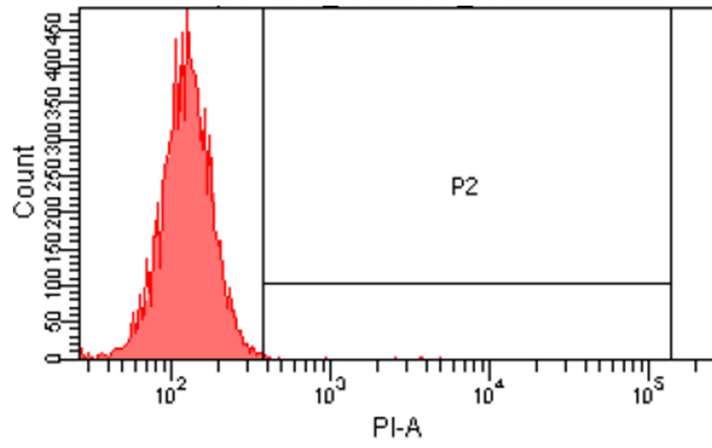

n LIP

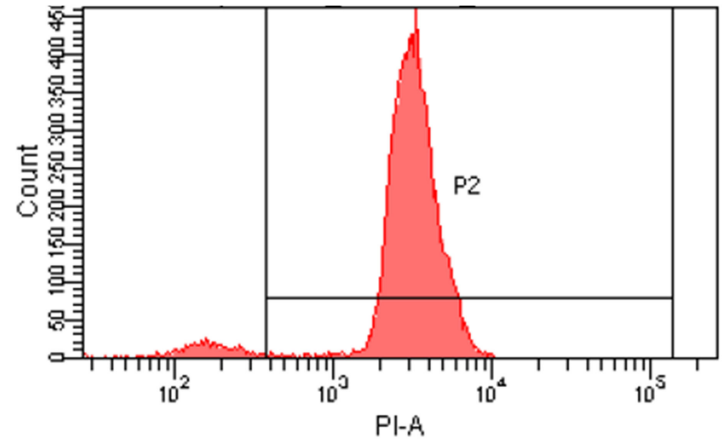

r LIP

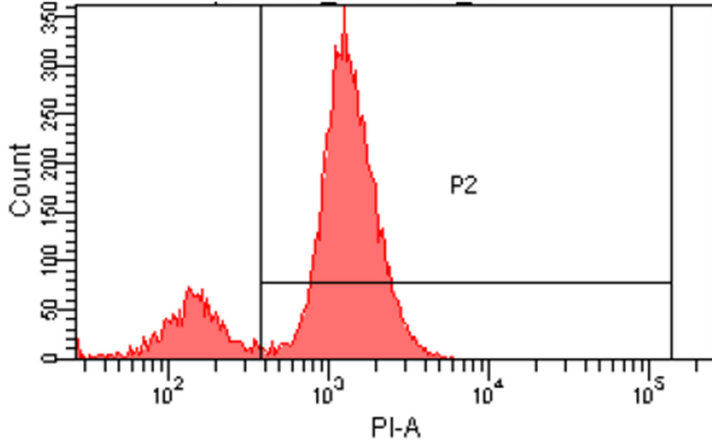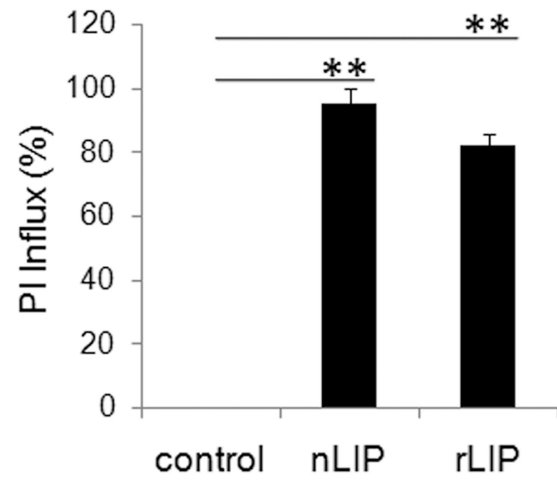

Supplement: Supplementary file 4 — The cytotoxicity of native LIP and recombinant LIP detected by flow cytometry. The MCF-7 cells were incubated with native LIP and recombinant LIP at 37 °C for 12 h. Cell death was analyzed by PI staining and by flow cytometry. Untreated cells were used as a negative control. All experiments were repeated at least three times with similar results. (PDF 904 kb) [file 12964_2017_198_MOESM2_ESM.pdf]

# Supplement Figure 3 Y Pang

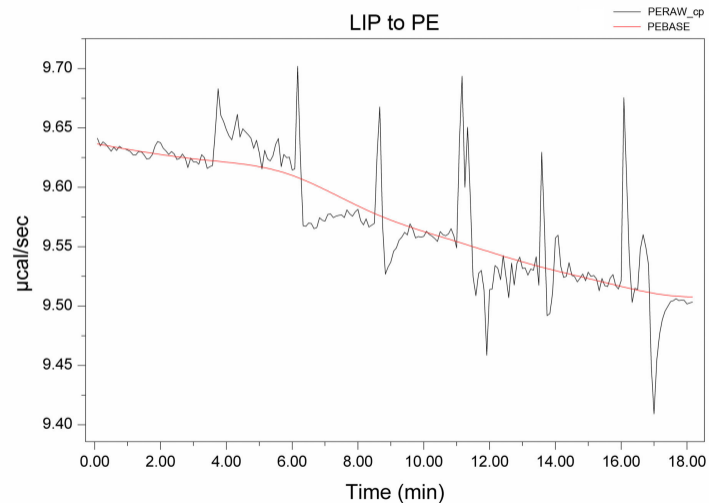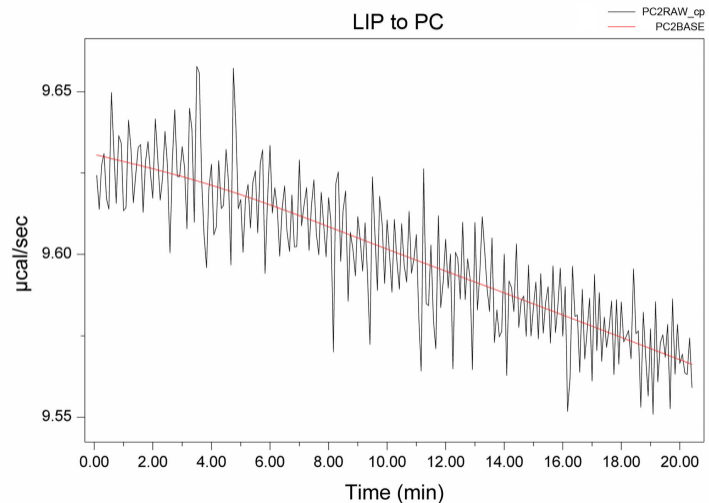

Supplement: Supplementary file 5 — Calorimetric measurements of the LIP interaction with PE/PC. Calorimetric measurements of the LIP interaction with PE and PC. (PDF 398 kb) [file 12964_2017_198_MOESM5_ESM.pdf]
